# The puppet interview to measure illness perceptions in paediatric oncology: development and psychometric properties in acute treatment and follow-up care

**DOI:** 10.1186/s12887-024-04586-5

**Published:** 2024-02-13

**Authors:** Kristina Herzog, Florian Schepper, Jessy Herrmann, Julia Martini

**Affiliations:** 1 Department of Psychiatry and Psychotherapy, Faculty of Medicine of the Technische Universität Dresden, Fetscherstr. 74, Dresden, 01307 Germany; 2 Elternhilfe Für krebskranke Kinder Leipzig e.V., Leipzig, Germany; 3 Department of Paediatric Oncology, Haematology, and Haemostaseology, Leipzig University, Leipzig, Germany; 4 Department of Paediatric Psychiatry, Psychotherapy, and Psychosomatics, Leipzig University, Leipzig, Dresden, Germany

**Keywords:** Cancer, Common-Sense-Model, Diagnostic testing, Illness perceptions, Paediatrics, Psycho-oncology, Puppet interview

## Abstract

**Background:**

Illness perceptions comprise assumptions about symptoms, timeline, consequences, controllability, and emotional responses to an illness. Recent evidence shows that illness perceptions are associated with coping and well-being. So far, assessment in paediatric care was based on parental report only, since no instrument for the direct assessment of young children was available. We aim to describe the development (incl. indication and contraindication) of an innovative puppet interview to assess illness perceptions in children with cancer from the age of four years. Moreover, we investigate longitudinal trajectories and examine psychometric properties.

**Methods:**

The puppet interview was developed based on the Illness-Perception-Questionnaire-Revised and the Berkeley-Puppet-Interview. Longitudinal trajectories and psychometric properties were examined in a sample of patient-parent dyads (*N* = 75) in a prospective longitudinal study in acute treatment with a 1-year follow-up (study 1: *n*_*T1*_ = 41, *n*_*T2*_ = 27) and in a cross-sectional study in follow-up care (study 2, *n* = 34).

**Results:**

The puppet interview is comprehensible and well-received by children in acute treatment and follow-up care. There were significant differences in perceptions of a chronic timeline (*U* = 301.00, *p* = .008), consequences (*U* = 251.00, *p* = .008), and emotional representations (*U* = 244.50, *p* = .020) between children in acute treatment and children in follow-up care. Over the course of one year, children in acute treatment perceived more symptoms as part of their illness (*M*_*T1*_ = 3.6, *SD*_*T1*_ = 2.9, *M*_*T2*_ = 4.5, *SD*_*T2*_ = 3.1, *n* = 27, *Z* = -2.603, *p* = .009). We found expected intercorrelations between illness perception dimensions, e.g. between perception of consequences and emotional representations (*r*_*τ*_ = .27, *p* = .033), and between perception of a chronic timeline and consequences (*r*_*τ*_ = .38, *p* = .001). Moreover, we found confirming results regarding construct validity, as child’s perceptions of symptoms correlated with their self-rated HRQoL (*r*_*τ*_ = -.32, *p*_*adj.*_ = .014). Also parent-rated subscale on illness-specific aspects of child’s HRQoL correlated with child’s perception of symptoms (*r*_*τ*_ = -.26, *p*_*adj.*_ = .016), cyclicity (*r*_*τ*_ = -.28, *p*_*adj.*_ = .016), and consequences (*r*_*τ*_ = -.34, *p*_*adj.*_ = .014). Acceptable internal consistency was shown for the dimensions timeline-acute/chronic and personal control.

**Conclusions:**

Parental report can now be complemented by a self-report of illness perceptions in children aged four years and older. This will allow for the further adaptation of medical and psychosocial care during and after acute cancer treatment.

**Trial registration:**

The study has been pre-registered at the German Clinical Trials Register (registered 30/06/2020; DRK-S00022034) and at the Open Science Foundation (https://osf.io/7xr6z).

**Supplementary Information:**

The online version contains supplementary material available at 10.1186/s12887-024-04586-5.

## Background

The diagnosis of an illness, such as cancer, is associated with various psychological problems during and after acute treatment. The individual perceptions and beliefs about the illness seem to be important for individual illness management [1].

**Fig. 1:**
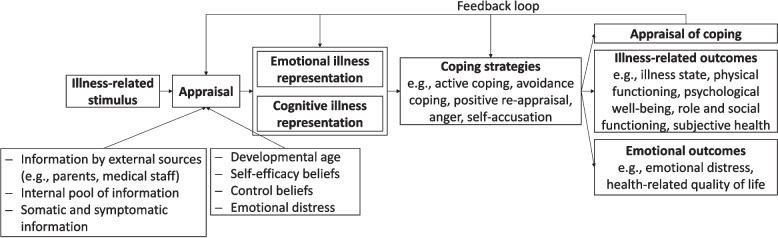
Schematic representation of Leventhal et al.’s CSM [2] under consideration of individual influencing factors [31]

The Common-Sense Model of Illness Representation (CSM) [2] is an influential theoretical model that links individual illness management and coping strategies with their respective health behaviour. The model postulates that individuals create cognitive representations of their illness when being confronted with it. These *illness perceptions* are based on information accessible to them (e.g. from conversations with others, education by medical personnel, experience of the illness) and individual differences (e.g. age, self-efficacy beliefs, control beliefs, optimism) and they determine the self-regulation process in dealing with the illness, for example the emotional response and coping style [3]. Individual illness perceptions thus may have an indirect influence on the course of the illness, for example via medication adherence [4]. For a schematic presentation of the CSM including potential influencing factors, see Fig. 1. According to the CSM, illness perceptions include the following dimensions: (1) Identity, i.e. the name and symptoms associated with the illness, (2) perceived cause of the illness or the symptoms, (3) perceived consequences, (4) perceived personal and treatment control, (5) perceived chronicity and cyclicity, (6) perceived comprehensibility, and (7) emotions associated with the illness (e.g. sadness, fear, agitation).

Illness perceptions can be assessed with the *Illness Perception Questionnaire* (IPQ) [5] and its revised version (IPQ-R) [6]. The IPQ-R is an internationally recognized instrument for assessing illness perceptions with 64 items on the following dimensions, in line with the CSM:

1. Illness identity (i.e. symptoms)
2. Timeline (acute/chronic, cyclical)
3. Consequences
4. Personal control
5. Treatment control (not included in the short German version by Gaab et al. [6])
6. Coherence
7. Emotional representations

The IPQ-R has been translated in numerous languages and has been used in over 100 studies [7] with various illnesses (e.g. cardiovascular illnesses, asthma, diabetes, and HIV) [4]. Questionnaire versions for relatives exist, for instance for spouses of patients with rheumatoid arthritis [8] or schizophrenia [9]. The IPQ-R has been predominantly used to examine adolescents and adults, but there is a lack of appropriate assessment methods for children. Also, parent’s proxy-report may not be useful as various studies show that while parents may reliably proxy-report on their child’s visible symptoms, their report may be less reliable for their child’s cognitive or emotional state [10–13]. In addition, the “ISPOR PRO Good Research Practices for the Assessment of Children and Adolescents Task Force” concluded that self-report is possible from about five years of age if the response format is adapted to the children’s age, though deductions in terms of psychometric properties may have to be made [14]. Moreover, letting the child self-report on their health may increase their sense of control. This highlights the importance of asking the children themselves about their perspectives.

Questionnaire versions for younger children exist, for example the shorter CIPQ for children with asthma or eczema from the age of 7 years [15] or the You-IPQ-R for adolescents with asthma from the age of 11 years [16]. However, psychometric evaluation of the CIPQ is limited and the questionnaire is based on the IPQ which has since then undergone major revisions. Concerning the You-IPQ-R, Heyduck-Weides and colleagues found internal consistency to be lower when younger children under the age of 14 years were examined. They concluded that “the complexity and readability of the You-IPQ-R may present a challenge to younger adolescents thus limiting its appropriateness and applicability in very young patients” ([16]; p. 18). This highlights the importance of using alternative methods to assess illness perceptions in younger children.

The aim of this article is to describe the development and application of the IPQ-R-Puppet Interview as an innovative age-appropriate method to assess illness perceptions in 4–11-year-old children in paediatric oncology. The development of the German puppet interview and first psychometric results (pilot study: *n* = 11, main study: *n* = 64 children in acute treatment or follow-up care) have been published by Schepper et al. [17]. In the pilot study, construct validity has been examined: The more chronic children perceived their illness and the more negative consequences they expected, the lower they rated their internal control (IE-4 [18]; *r* = -0.71, *p* ≤ 0.05; *r* = -0.62, *p* ≤ 0.05). Perception of a cyclical trajectory correlated with higher emotional strains (SDQ [19]; *r* = 0.66, *p* ≤ 0.05). In the main study, results on acceptance and comprehensibility of the puppet interview were good (“I enjoyed our conversation a lot.”: 89.1%; “I understood all questions well.”: 82.8%). Internal consistency of the dimensions “timeline-acute/chronic” (*α* = 0.75) and “personal control” (*α* = 0.72) was acceptable, whereas internal consistency for the other dimensions was lower.

In order to make the puppet interview available to the international research community, its development and results on psychometric properties from a larger longitudinal sample (*N* = 75 paediatric patients and their parents) are presented here (see also COPE Guidelines [20]). Research objectives for our study were (1) the age-appropriate adaptation of the IPQ-R as a puppet interview for 4–11-year-old children in paediatric oncology including (contra-) indications and recommendations to manage critical situations and (2) psychometric testing of the IPQ-R-Puppet Interview.

## Methods

### Participants and design

For the puppet interview, the approximate age range was set to 4–11 years. Younger children may have trouble understanding the interview questions, while older children may feel more comfortable completing a questionnaire. Within the larger research project, older children were also given the opportunity to participate in the puppet interview instead of a questionnaire. All children above the age of 11 years opted to complete the questionnaire with a researcher (KH) being available to answer any questions.

In study 1, children aged 4–11 years in acute treatment and a parent were recruited in the acute wards for paediatric oncology at the university hospitals in Dresden and Leipzig (Germany) and examined in a prospective-longitudinal observational study with two assessment points (baseline and one-year follow-up). All families who met the inclusion criteria and who were in acute treatment at the time of recruitment (10–11/2019 and 06/2020–10/2021) were asked to participate.

In study 2, 4–11-year-old children in follow-up care and a parent were recruited between 06–12/2020 in aftercare in Dresden and Leipzig (Sonnenstrahl e.V. Dresden, Elternhilfe e.V. Leipzig) for an observational cross-sectional study. All families who met the inclusion criteria were asked to participate.

Inclusion criteria were (1) patients aged 4 to 11 years with any oncological diagnosis and one parent, (2) for children in acute treatment (study 1): first diagnosis at least one month ago, (3) for children in follow-up care (study 2): first diagnosis at least two years ago; being off active treatment. Exclusion criteria were (1) inability to understand the puppet interview (e.g. due to language barriers or cognitive impairment; assessed by psychosocial staff), and (2) palliative care.

Ethics approval was obtained from the ethics committee of the Technische Universität Dresden (EK-514112015) and the Universität Leipzig (366/14-ff). Written informed consent was obtained from the parents of all participating children and the children themselves. The study has been pre-registered at the German Clinical Trials Register (DRKS-00022034) and at the Open Science Foundation (https://osf.io/nmu5e).

### Procedure and measures

The puppet interview was developed based on the Illness Perception Questionnaire – Revised (IPQ-R) and the Berkeley Puppet Interview (see below, development of the puppet interview).

To examine research questions 1 and 2, all children participated in the puppet interview. The puppet interview was administered based on an interview guide (Appendix 1) by one of the authors (KH), who is a clinical psychologist. KH has been trained in the administration of the puppet interview by FS, an experienced psycho-oncologist and expert in play therapy. The interviews typically took place in the child’s room at the hospital or in a room the parent’s association. The interviews took approximately 15 min (maximum 30 min including play breaks) and were recorded with a video camera and then transcribed by KH.

While the children participated in the puppet interview, parents (generally the main caregiver) completed questionnaires concerning their child’s internal control (IE-4 [18]), general self-efficacy beliefs (ASKU [21]), optimism (SOP-2 [22]), and emotional problems (SDQ [19]). Children and parents also completed questionnaires on the child’s health-related quality of life (KINDL-R [23]). The interviewer (KH) was available to answer any questions by the children or parents.

### Development and implementation of the puppet interview

#### Child-specific adaptation of the IPQ-R

Given that a playful approach to diagnostics and treatment is often the method of choice when working with children [24], we adapted the IPQ-R based on the Berkeley Puppet Interview (BPI [25]). In the BPI, two identical hand puppets are used to make opposing statements about themselves and then ask the child to decide which statement fits them best (e.g. “I have lots of friends.” – “I don’t have many friends. How about you [name]?”). The BPI has been used in various studies for children aged 4–8 years. Puppet interviews have important advantages when interviewing children: Puppets allow an age-appropriate interview in which the puppets interact with the child and can adapt to their language level. Also, due to its playful character, the puppet interview may be less stressful for the child than a questionnaire.

**Fig. 2:**
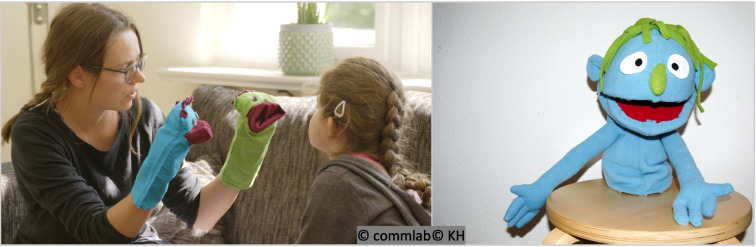
Left: Using two hand puppets in a diagnostic puppet interview. Right: Another example of a self-made hand puppet

The adaptation of the short German version of the IPQ-R [6] as a puppet interview takes into account the linguistic and cognitive development of children aged 4 to 11 years [25, 26]. To achieve this, the 5-point Likert items of the IPQ-R were dichotomized and presented as opposing statements by two puppets “Beppi” and “Seppi” [25, 27]. The child is asked to indicate either verbally or by tapping which puppet presented the most appropriate answer. Table 1 shows the dimensions and statements of the two puppets in the puppet interview. The dimension “treatment control” was added later to the puppet interview as it was not part of the short German version of the IPQ-R that we used as a basis. The items for this dimension were adapted from the IPQ-R [28]. The symptoms-dimension includes a list of 14 symptoms and the puppets ask about the presence of a symptom and whether the child believes that this symptom was caused by their illness or treatment (“I believe that [the symptom] happened/did not happen because of your cancer illness or treatment.”). The remaining dimensions (timeline-acute/chronic, timeline-cyclical, consequences, personal control, treatment control, coherence, emotional representations) include three items per dimension. To avoid response tendencies, items are not presented dimension by dimension, as in the IPQ-R. For the same reason, and to avoid identification with one of the puppets (e.g. with the puppet that reports fewer negative experiences), the items are assigned to the puppets in a randomized way. Scores for the dimensions are calculated by summing the items after reverse scoring. Scores for the symptoms dimension range from 0 to 14, while scores for the other dimension range from 0 to 3. Higher scores indicate more associated symptoms, more negative perception of chronicity, cyclicity, and emotional representations, and more positive perception of personal control, treatment control, and illness coherence. The sewing instructions for the hand puppets are available online on our website (https://tud.link/b2ys), so making the puppets is easy and inexpensive. An example setup of this diagnostic puppet interview is shown in Fig. 2. An interview guide is provided in Appendix 1. Due to the standardized instructions and closed response format the objectivity of implementation and analysis of the puppet interview is judged high [29].

**Table 1:**
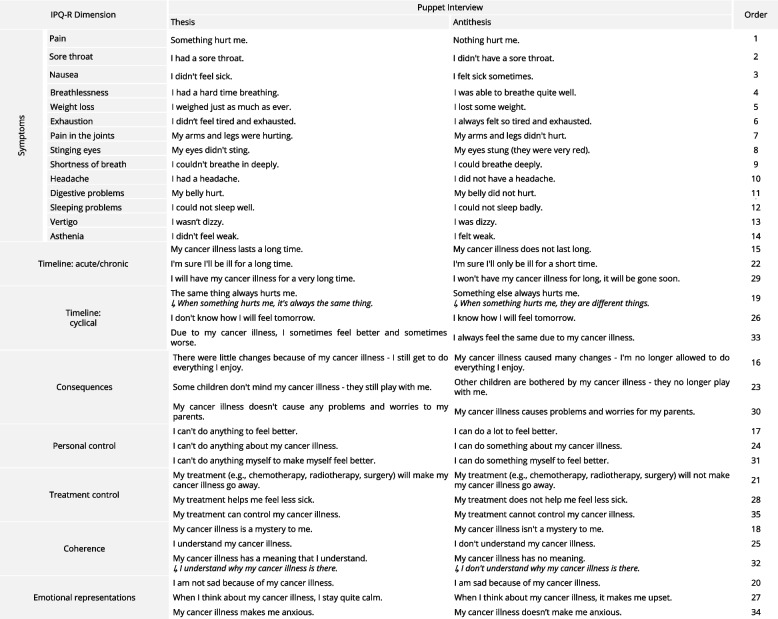
Item adaptation of the IPQ-R as a puppet interview. Thesis and antithesis are presented randomly by two hand puppets. Some items have been reworded because they were not well understood by a significant number of children. This table presents both the original as well as the reworded versions of the items

Thesis and antithesis are presented randomly by two hand puppets. Some items have been reworded because they were not well understood by a significant number of children. This table presents both the original as well as the reworded versions of the items in italics

### Conducting the interview

Preparation: Interviewers should have experience in the psychosocial treatment of children (e.g. psychologist). According to our practical experience, it is imperative to practice the puppet interview before using it. For example, eye contact between the interviewer and the child would interrupt the child's interaction with the puppets and the child might enter a dialogue with the interviewer instead of the puppets. We recommend practicing the puppet interview about three times in order to develop sufficient confidence in handling the puppets.

Conducting the interview: Before the start of the interview, the child and their parents are informed about its content, and the child should be asked if they agree to be asked questions by two puppets. The puppets could then be introduced as co-workers of the interviewer or as former patients. Parents should ideally not be present for the interview as this might distract the child. Some children, however, may feel more comfortable with the parents being present. In this case, it is recommended that the parents stay outside of their child’s field of vision and occupy themselves. Parents should furthermore be instructed not to answer on behalf of their child or intervene in the interview in any other way. At the beginning of the interview, it is important to arouse the child’s curiosity. For example, the puppets might tell a funny/intriguing story or ask open-ended, resource-oriented questions about hobbies, favourite films, things they have in common, etc. to create a playful atmosphere. Many children also ask the puppets questions (e.g. “Where are your parents?”, “Why is one of you blue and the other green?”), Similarities between the puppets and the child (e.g. same age) are useful in building a relationship. Then, the cover story of the puppet interview is told: Beppi and Seppi explain that they have also spent some time in hospital due to severe illness and that they want to talk about their experiences. Then, the puppets inquire about how the child is currently experiencing their illness. Throughout the interview, the child’s and the puppets’ conversational portions should be well-balanced. To build a relationship, introductory (“You know what, [name]?”), probing (“What do you think?”, “What is it like for you?”), and validating (“So you felt the same as I do.”) phrases can be used. Moreover, children may benefit from the puppet reflecting the child’s emotions. At all times, it is important that the interviewers respond emphatically, appreciatively, and genuinely. Differences in style for different age groups may be applied, e.g. in terms of language outside of the standardized items or in terms of interests or hobbies of the puppets.

The following examples are provided to illustrate how to manage common interview situations ant the beginning:

- Example 1– Arousing the child’s curiosity: At first, 6-year-old R. was wary of meeting the interviewer and the puppets. She did not want to tell the puppets her name, but nevertheless appeared to be very curious about them. The puppets made a game of guessing R.’s name and telling jokes, thereby engaging R. into a playful atmosphere. In the end, the puppets figured out R.’s name and from this moment, the ice was broken. R. started to play animatedly with the puppets and responded enthusiastically to their suggestion to talk about their illnesses. She often cuddled the puppets or offered them some of her food and drink.
- Example 2 – Negotiating “rules”: 7-year-old D. was very curious about the puppets and enthusiastically “woke” them with the interviewer. He then immediately began telling the puppets about his day and invented a song about the puppets. The puppets suggested that D. answer a few questions and then sing with the puppets for a few minutes. In this way, the puppet interview could be carried out completely.
- Example 3 – Building a relationship: 5-year-old M. was very shy at the beginning of the appointment for the puppet interview. She hid behind her father and did not want to talk with the hand puppets. The interviewer therefore began a conversation with M.’s father. The father explained that M. had had a stressful day with many medical appointments. In order to establish contact, the interviewer then asked about M.’s interests and hobbies. Over the course of the conversation, M. became curious and sat next to the interviewer instead of hiding behind her father to join into the conversation. To build a relationship, the interviewer asked M. if she wanted to paint a picture together with the interviewer. M. agreed enthusiastically and took out her painting utensils. At the end of the appointment, she agreed to meet the two hand puppets again later and suggested to paint together with the puppets. At the second appointment, M. was excited about the prospect of painting together and the puppet interview could be conducted completely.

Indications for the puppet interview are summarized in Table 2, together with potential difficulties and how they can be met.

**Table 2:** Indications and contextual factors of the puppet interview

Setting	Acute treatment, follow-up care
(Developmental) age, cognitive development	4–11 years
Language	Requirements are comparatively low; whether they are met can be evaluated through play
Contextual factors	commotion at the adjacent bed at the hospital tiredness due to therapy side effects of the illness or treatment (e.g. pain, fever, nausea) important medical examinations are scheduled, or the child and parents are upset or anxious by inconclusive findings or pending test results Such situations do not necessarily contradict the use of the puppet interview. Sometimes, especially in acute crisis, the puppet interview offers a welcome diversion from the daily routine of the clinic and treatment, or the next treatment steps can be discussed in a playful way. Many children engage in conversation with the puppet even despite severe nausea or vomiting in order to talk about this. This shows that the puppet interview can also be useful in acute treatment phases
Motivational factors and concentration	concentration for the entire duration of the puppet interview (especially younger children) wariness of the (unfamiliar) interviewer If the child is unfocussed, the puppets can negotiate “rules” for the puppet interview. Breaks should be incorporated during which the child can play with the puppets of tell them what they have experienced in the previous days. A first appointment can be used to build a relationship with the child (e.g. by drawing or playing) before conducting the interview

## Statistical analysis

Known-groups validity: To analyse how illness perceptions differ between treatment phases, Mann–Whitney *U*-tests were used to compare illness perceptions of patients in acute treatment with those of patients in follow-up care. The dimensions timeline-acute/chronic, timeline-cyclical, consequences, personal control, treatment control, coherence, and emotional representations refer to the child’s current perception. The symptoms-dimension, on the other hand, refers to any symptoms that have been perceived at any point during the illness/treatment (retrospectively).

Longitudinal trajectories of illness perceptions over the course of one year of children in acute treatment were calculated using Wilcoxon signed-rank tests.

Acceptance and comprehensibility ratings for the IPQ-R-Puppet Interview were analyzed in percentages.

Construct validity: Do the dimensions of the IPQ-R-Puppet Interview correlate with external constructs according to our hypotheses (Kendall-Tau correlations)? To account for multiple comparisons, the Benjamini–Hochberg method will be used to calculate adjusted *p*-values. Based on psychometric findings on the German IPQ-R and the You-IPQ-R [16, 30], as well as theoretical considerations of the CSM [4, 31] we expect internal locus of control to correlate with the personal control-dimension, self-efficacy beliefs and optimism with the emotional representations-dimension, emotional problems with the symptoms-, consequences-, and emotional representations-dimensions, and health-related quality of life (HRQoL) with the symptoms-, timeline-, consequences-, and emotional representations-dimensions. Internal locus of control was proxy-rated by parents using a four-item scale (IE-4 [18]). Self-efficacy beliefs were proxy-rated by parents using a three-item scale (ASKU [21]). Optimism was proxy-rated by parents using a single-item scale (SOP [22]). The IE-4, ASKU, and SOP scales were adapted for the parent’s proxy-report (e.g. “my child” instead of “I”). Emotional problems were proxy-rated by parents with the “emotional problems” subscale of the Strength and Difficulties Questionnaire (SDQ) that includes items on anxiousness and depressiveness [19] which have been shown to be associated with illness perceptions in previous studies [32]. HRQoL was self-and proxy-rated using the KINDL-R scale [23]. Parent’s proxy-ratings included subscales on physical and emotional well-being, self-esteem, family, friends, daily functioning (school, pre-school), and illness-related well-being that have been shown to be associated with illness perception in previous studies [33].

Interscale correlations: Do the dimensions of the IPQ-R-Puppet Interview intercorrelate according to our hypotheses (Kendall-Tau correlations)? Based on results from studies on psychometric properties of the English and German IPQ-R in adult patients with different illnesses, and the German You-IPQ-R for adolescents with asthma [16, 28, 30] we expect the following interscale correlations: More negative emotional representations correlate with perception of more symptoms, a more chronic and cyclical timeline, perception of more negative consequences, lower coherence, and lower personal control. Additionally, we expect the timeline-dimension to correlate with each other and with the perception of more negative consequences. The perception of more symptoms correlates with the perception of more negative consequences, and the perception of higher coherence correlates with the perception of higher personal control over the illness.

Internal consistency of the items for each dimension was assessed with the Kuder-Richardson-20 formula for dichotomous items.

Missing data: Data of 53.3% of cases was complete. For the statistical analyses, listwise deletion method was used.

Analyses were performed using IBM SPSS Statistics 27.0. For all tests, *p* < 0.05 was considered statistically significant.

## Results

### Sample

**Fig. 3:**
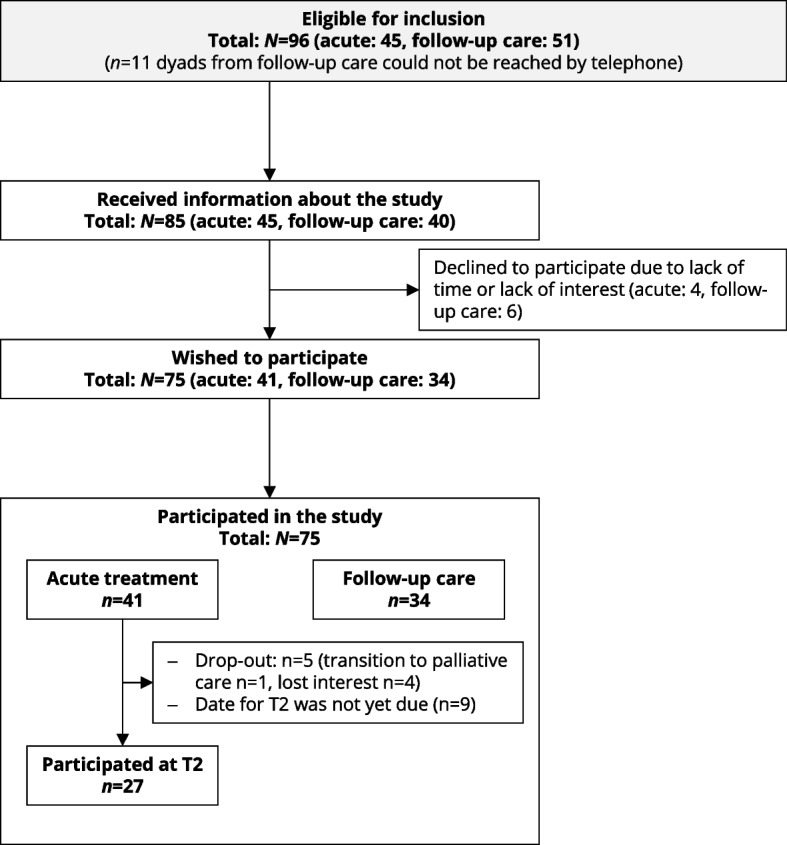
Flow chart on participation

From June 2020 to December 2022,* N* = 75 child-parent-dyads were interviewed (acute treatment: *n*_*T1*_ = 41, *n*_*T2*_ = 27; follow-up care: *n* = 34) in total (Fig. 3). For the study in acute treatment, therefore, the current response rate was 65.9% with data collection for T2 still being underway at time of analysis. Demographic and medical information are summarized in Table 3.

**Table 3:** Sample characteristics

	Study 1 (acute treatment, baseline) (*n* = 41)	Study 2 (follow-up care) (*n* = 34)
Gender (*n*, %)		
male	17 (41.5)	22 (64.7)
female	24 (58.5)	12 (35.3)
diverse	0 (0.0)	0 (0.0)
Age (*M, SD*)	6.93 (2.27)	7.94 (2.28)
Diagnosis (*n*, %)		
Leukaemia	20 (48.8)	11 (32.4)
Lymphoma	2 (4.9)	2 (5.9)
Tumour of the central nervous system	11 (26.8)	8 (23.5)
Other solid tumour^a^	8 (19.5)	11 (32.4)
Langerhans cell histiocytosis	0 (0.0)	2 (5.9)
Time since diagnosis in months (*M, SD*)	8.03 (16.93)	56.13 (24.88)
Treatment modality (*n*, %—*multiple responses possible)*		
Chemotherapy	36 (90.0)	28 (82.4)
Radiotherapy	5 (12.5)	4 (11.8)
Surgical measures	14 (35.0)	14 (41.2)
Haematopoietic stem cell transplant	0 (0.0)	0 (0.0)
Other^b^	0 (0.0)	2 (5.9)

^a^e.g. Ewing sarcoma, Hepatoblastoma

^b^e.g. immune therapy, BRAF inhibitor

### Descriptive results (*n* = 75) and known group validity

**Fig. 4:**
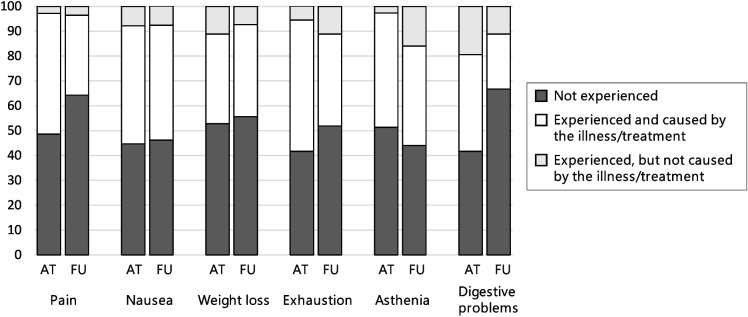
Descriptive comparison of perceptions of the most commonly experienced symptoms in patients in acute treatment (*n* = 41), compared with patients in follow-up care (*n* = 34) based on the IPQ-R Puppet interview. Note: In follow-up care, questions on symptoms refer retrospectively to the time during acute treatment

**Fig. 5:**
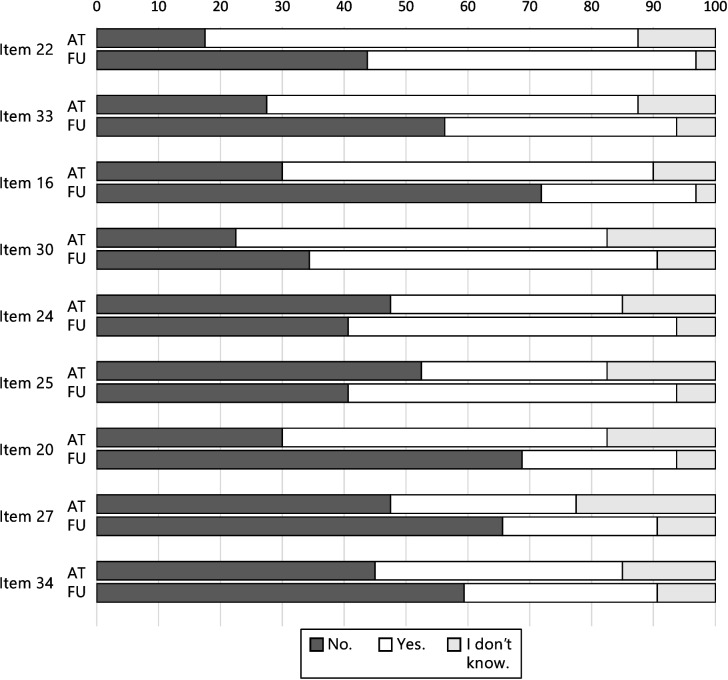
Descriptive comparison of selected items of the other dimensions in patients in acute treatment (*n* = 41), compared with patients in follow-up care (*n* = 34). Note: The statements refer to the child’s current perception. Item 22 (timeline-acute/chronic): “I’m sure I’ll be ill for a long time.”, item 33 (timeline-cyclical): “Due to my cancer illness, I sometimes feel better and sometimes worse.”, item 16 (consequences): “My cancer illness caused many changes – I’m no longer allowed to do everything I enjoy.”, item 30 (consequences): “My cancer illness causes problems and worries to my parents.”, item 24 (personal control): “I can’t do anything about my cancer illness.”, item 25 (coherence): “I don’t understand my cancer illness.”, item 20 (emotional representations): “I am sad because of my cancer illness.”, item 27 (emotional representations): “When I think about my cancer illness, it makes me upset.”, item 34 (emotional representations): “My cancer illness makes me anxious”

**Fig. 6:**
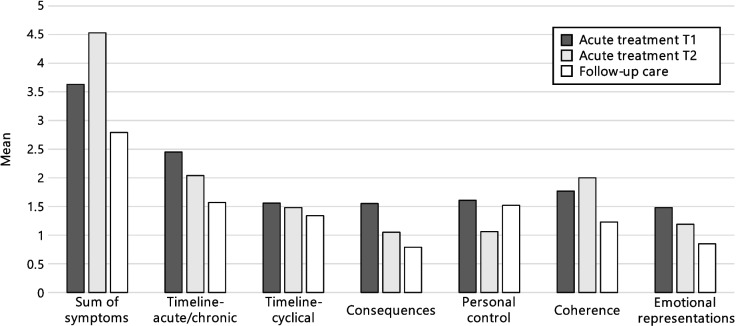
Descriptives of illness perception scores in patients in acute treatment at two time points one year apart (longitudinal study 1: *n*_*T1*_ = 41, *n*_*T2*_ = 27) and patients in follow-up care (cross-sectional study 2: *n* = 34). Note. Scores for the symptoms dimension range from 0 to 14, while scores for the other dimension range from 0 to 3. Higher scores indicate more associated symptoms, more negative perception of chronicity, cyclicity, and emotional representations, and more positive perception of personal control and illness coherence

Figure 4 (most common symptoms), Fig. 5 (selected items of the other dimensions), and Fig. 6 (scores of the dimensions) show that illness perceptions can differ depending on the treatment phase. Known group validity analyses showed significant differences between treatment phase (acute treatment vs. follow-up care) concerning the perception of chronicity (*U* = 301.00, *p* = 0.008), consequences (*U* = 251.00, *p* = 0.008), and emotional representations (*U* = 244.500, *p* = 0.020), with children in acute treatment perceiving their illness as more chronic, expecting more negative consequences, and experiencing more negative emotions in connection to the illness/treatment. A non-significant tendency could be observed for the coherence-dimension (*U* = 288.50, *p* = 0.058). No differences were found for the perception of symptoms (*U* = 458.50, *p* = 0.237), cyclical trajectory (*U* = 417.50, *p* = 0.268), and personal control (*U* = 392.50, *p* = 0.673),

### Longitudinal trajectories (*n*_*T2*_ = 27)

Descriptive results are shown in Fig. 6. When comparing illness perceptions at T1 and T2, significant differences in some dimensions are observed: Patients at T2 have experienced significantly more symptoms as part of their illness than at one year before at T1 (*M*_*T1*_ = 3.6, *SD *_*T1*_ = 2.9, *M *_*T2*_ = 4.5, *SD *_*T2*_ = 3.1, *n* = 27, *Z* = -2.603, *p* = 0.009). The other dimensions (timeline-acute/chronic, timeline-cyclical, personal control, coherence, emotional representations, consequences) did not change significantly over the course of one year, though a non-significant tendency could be observed for the consequences-dimension, with patients at T1 perceiving more negative consequences of their illness than they did one year later (*M*_*T1*_ = 1.6, *SD *_*T1*_ = 0.7, *M *_*T2*_ = 1.1, *SD *_*T2*_ = 0.7, *n* = 17, *Z* = -1.897, *p* = 0.058).

### Acceptance and comprehensibility

Acceptance of the puppet interview in the full sample was high (“I enjoyed our conversation a lot.”: 82.7%), though there were descriptive differences between age groups (aged 4–6 (*n* = 26) 73.1%; aged 7–8 (*n* = 24): 87.5%, aged 9–11 (*n* = 25): 82.7%).

Comprehensibility of the items in the puppet interview in the full sample was good (“I understood all questions well.”: 77.3%), though there were descriptive differences between age groups (aged 4–6 (*n* = 26): 65.4%; aged 7–8 (*n* = 24): 87.5%, aged 9–11 (*n* = 25): 80.0%). Follow-up inquiry showed that it was predominantly the same items that had been misunderstood in the interview. We reworded these items (Table 1).

### Construct validity

Results are summarized in Tables 4 and 5. The more symptoms a child perceived, the lower they self-reported on their overall HRQoL (*r*_*τ*_ = -0.32, *p*_*adj.*_ = 0.014). Moreover, significant associations were found between child’s illness perceptions and one parent-reported aspect of their child’s HRQoL, namely illness-related aspects (Table 5): Parents rated this to be lower for children who perceived more symptoms, a more cyclical trajectory, and more negative consequences (*r*_*τ*_ = -0.26, *p*_*adj.*_ = 0.016; *r*_*τ*_ = -0.28, *p*_*adj.*_ = 0.016; *r*_*τ*_ = -0.34, *p*_*adj.*_ = 0.014).

**Table 4:** Construct validity in the main study (*N* = 75): Kendall Tau correlations *(r*_*τ*_*, p)* between the dimensions of the IPQ-R Puppet Interview and parent-reported measures for beliefs about internal control, general self-efficacy, optimism, emotional problems, and self- and parent-reported child’s health-related quality of life

					Parent-report						Self-report	
	Internal control belief (IE-4)		Self-efficacy belief (ASKU)		Optimism (SOP)		Emotional problems (SDQ)		HRQoL (KINDL-R)		HRQoL (KINDL-R)	
	*r*_*τ*_* (p*_*unadj.*_*)*	*r*_*τ*_* (p*_*adj.*_*)*	*r*_*τ*_* (p*_*unadj.*_*)*	*r*_*τ*_* (p*_*adj.*_*)*	*r*_*τ*_* (p*_*unadj.*_*)*	*r*_*τ*_* (p*_*adj.*_*)*	*r*_*τ*_* (p*_*unadj.*_*)*	*r*_*τ*_* (p*_*adj.*_*)*	*r*_*τ*_* (p*_*unadj.*_*)*	*r*_*τ*_* (p*_*adj.*_*)*	*r*_*τ*_* (p*_*unadj.*_*)*	*r*_*τ*_* (p*_*adj.*_*)*
Symptoms associated with the illness	.13 (.186)	.13 (.651)	-.04 (.654)	-.04 (.763)	-.08 (.428)	-.08 (.885)	.14 (.148)	.14 (.851)	-.10 (.269)	-.10 (.904)	**-.32 (.002)**	**-.32 (.014)**
Timeline-acute/chronic	.10 (.377)	.10 (.773)	-.14 (.198)	-.14 (.630)	**-.24 (.026)**	-.24 (.182)	.04 (.729)	.04 (.851)	-.06 (.548)	-.06 (.904)	-.21 (.066)	-.21 (.154)
Timeline-cyclical	**.24 (.037)**	.24 (.259)	.13 (.270)	.13 (.630)	-.18 (.117)	-.18 (.410)	.06 (.612)	.06 (.851)	-.02 (.867)	-.02 (.961)	**-.26 (.022)**	-.26 (.077)
Personal control	.05 (.666)	.05 (.773)	-.04 (.741)	-.04 (.763)	.01 (.922)	.01 (.922)	.10 (.384)	.10 (.851)	.08 (.432)	.08 (.904)	.04 (.742)	.04 (.742)
Coherence	-.07 (.529)	-.07 (.773)	.16 (.142)	.16 (.630)	.05 (.632)	.05 (.885)	-.05 (.690)	-.05 (.851)	.01 (.961)	.01 (.961)	-.09 (.482)	-.09 (.562)
Consequences	-.05 (.678)	-.05 (.773)	-.04 (.745)	-.04 (.763)	.05 (.631)	.05 (.885)	.02 (.859)	.02 (.859)	-.10 (.363)	-.10 (.904)	-.20 (.093)	-.20 (.163)
Emotional representations	-.03 (.773)	-.03 (.773)	.03 (.763)	.03 (.763)	.03 (.790)	.03 (.922)	-.10 (.373)	-.10 (.851)	.05 (.646)	.05 (.904)	-.09 (.429)	-.09 (.562)

To account for multiple comparisons, the Benjamini–Hochberg adjustment method was used (false discovery rate; *k* = 7). *p*_*unadj.*_ indicates p-values before adjustment, while *p*_*adj.*_ indicates *p*-values after adjustment. Bold characters indicate a significant result (*p* < .05). As the dimension “treatment control” was added later to the puppet interview, no information on construct validity is available yet. Measures for internal control ^[IE−4; 18]^, general self-efficacy ^[ASKU; 21]^, optimism ^[SOP−2; 22]^, emotional problems ^[SDQ; 19]^, and self- and parent-reported child’s health-related quality of life ^[KINDL−R; 23]^

**Table 5:** Construct validity in the main study (*N* = 75): Kendall Tau correlations *(r*_*τ*_*, p)* between the dimensions of the IPQ-R Puppet Interview and parent-reported measures for different dimensions of the child’s health-related quality of life ^[KINDL−R; 23]^

	HRQoL													
	Physical well-being		Emotional well-being		Self-esteem		Family		Friends		Daily functioning (school, pre-school)		Illness-related well-being	
	*r*_*τ*_* (p*_*unadj.*_*)*	*r*_*τ*_* (p*_*adj.*_*)*	*r*_*τ*_* (p*_*unadj.*_*)*	*r*_*τ*_* (p*_*adj.*_*)*	*r*_*τ*_* (p*_*unadj.*_*)*	*r*_*τ*_* (p*_*adj.*_*)*	*r*_*τ*_* (p*_*unadj.*_*)*	*r*_*τ*_* (p*_*adj.*_*)*	*r*_*τ*_* (p*_*unadj.*_*)*	*r*_*τ*_* (p*_*adj.*_*)*	*r*_*τ*_* (p*_*unadj.*_*)*	*r*_*τ*_* (p*_*adj.*_*)*	*r*_*τ*_* (p*_*unadj.*_*)*	*r*_*τ*_* (p*_*adj.*_*)*
Symptoms associated with the illness	**-.20 (.032)**	-.20 (.224)	**-.20 (.034)**	-.20 (.238)	**-.19 (.044)**	-.19 (.308)	.02 (.852)	.02 (.959)	.08 (.425)	.08 (.595)	.02 (.828)	.02 (.828)	**-.26 (.007)**	**-.26 (.016)**
Timeline-acute/chronic	-.07 (.522)	-.07 (.913)	-.10 (.354)	-.10 (.620)	-.05 (.649)	-.05 (.975)	.14 (.194)	.14 (.959)	-.06 (.555)	-.06 (.648)	-.06 (.571)	-.06 (.666)	-.15 (.164)	-.15 (.230)
Timeline-cyclical	-.17 (.111)	-.17 (.389)	-.11 (.298)	-.11 (.620)	-.03 (.762)	-.03 (.975)	.01 (.932)	.01 (.959)	.09 (.419)	.09 (.595)	.08 (.456)	.08 (.666)	**-.28 (.007)**	**-.28 (.016)**
Personal control	.01 (.913)	.01 (.913)	.03 (.790)	.03 (.790)	.00 (.975)	.00 (.975)	.02 (.844)	.02 (.959)	.15 (.179)	.15 (.595)	.17 (.134)	.17 (.560)	-.04 (.708)	-.04 (.708)
Coherence	-.10 (.340)	-.10 (.793)	-.04 (.742)	-.04 (.790)	-.01 (.961)	-.01 (.975)	.05 (.655)	.05 (.959)	.01 (.930)	.01 (.930)	.10 (.375)	.10 (.666)	-.13 (.218)	-.13 (.254)
Consequences	.02 (.863)	.02 (.913)	-.14 (.205)	-.14 (.620)	-.16 (.163)	-.16 (.571)	-.10 (.403)	-.10 (.959)	-.10 (.379)	-.10 (.595)	-.08 (.483)	-.08 (.666)	**-.34 (.002)**	**-.34 (.014)**
Emotional representations	-.02 (.871)	-.02 (.913)	.05 (.626)	.05 (.790)	.04 (.738)	.04 (.975)	-.01 (.959)	-.01 (.959)	.11 (.322)	.11 (.595)	.17 (.160)	.17 (.560)	-.20 (.065)	-.20 (.114)

To account for multiple comparisons, the Benjamini–Hochberg adjustment method was used (false discovery rate; *k* = 7). *p*_*unadj.*_ indicates p-values before adjustment, while *p*_*adj.*_ indicates *p*-values after adjustment. Bold characters indicate a significant result (*p* < .05). As the dimension “treatment control” was added later to the puppet interview, no information on construct validity is available yet. Higher scores in the IPQ-R Puppet Interview indicate more associated symptoms, more negative perception of chronicity, cyclicity, and emotional representations, and more positive perception of personal control and ill-ness coherence. Higher scores in the KINDL-R indicate higher physical and emotional well-being, higher self-esteem, higher well-being concerning family and friends, better daily functioning in pre-school and school, and higher illness-related well-being

### Interscale correlations

Results are summarized in Table 6. The more symptoms the child perceived, the more severe illness-related consequences they expected (*r*_*τ*_ = 0.23, *p* = 0.036) and the more negative emotions they experienced (*r*_*τ*_ = 0.33, *p* = 0.003). A small correlation was found between perception of chronicity and perception of cyclicity (*r*_*τ*_ = 0.23, *p* = 0.044). Moreover, the more chronic the child perceived their illness, the more severe illness-related consequences they expected (*r*_*τ*_ = 0.38, *p* = 0.001). Lastly, the more negative consequences the child expected, the more negative emotions they experienced (*r*_*τ*_ = 0.27, *p* = 0.033).

**Table 6:** Kendall Tau correlations *(r*_*τ*_*, p)* between the dimensions of the IPQ-R Puppet Interview (*N* = 75)

		1	2	3	4	5	6	7
1	Symptoms associated with the illness	-						
2	Timeline-acute/chronic	.13 (.201)	-					
3	Timeline-cyclical	.15 (.153)	**.23** **(.044)**	-				
4	Personal control	.09 (.413)	-.05 (.696)	-.03 (.806)	-			
5	Coherence	.14 (.205)	-.06 (.610)	-.01 (.949)	.02 (.895)	-		
6	Consequences	**.23** **(.036)**	**.38** **(.001)**	.17 (.158)	.05 (.711)	.04 (.739)	-	
7	Emotional representations	**.33** **(.003)**	.21 (.081)	.06 (.606)	.00 (.984)	.11 (.346)	**.27** **(.033)**	-

Bold characters indicate a significant result (*p* < .05). As the dimension “treatment control” was added later to the puppet interview, no information on interscale correlations is available yet

### Internal consistency

Internal consistency of the dimensions of the puppet interview (three dichotomous items per dimension) was calculated using the Kuder-Richardson-20 formula (*N* = 75). Internal consistency of the dimensions “Timeline-acute/chronic” (α = 0.76) and “Personal control” (α = 0.75) was good. Lower internal consistency scores were found for the remaining dimensions (α_Timeline-cyclical_ = 0.10, α_Consequences_ = 0.29, α_Coherence_ = 0.55, α_Emotional representations_ = 0.44). As the dimension “treatment control” was added later to the puppet interview, no information on internal consistency is available yet.

## Discussion

In paediatric oncology, especially for very young patients, methods to assess patient-reported outcomes (PROs) are limited. This study presents a self-report method for the assessment of illness perceptions in 4–11-year-old children in acute cancer treatment or follow-up care. The puppet interview was developed based on the Illness-Perception-Questionnaire (IPQ-R) and the Berkeley-Puppet-Interview. Based on this, we examined longitudinal trajectories of illness perceptions and psychometric properties of the puppet interview.

Our results on differences between treatment groups show that children in acute treatment reported more negative perceptions concerning chronicity, consequences, and emotional representations than children in follow-up care. The diagnosis of children in follow-up care was on average four and a half years ago. Potentially, these children have few memories of their illness and hospital stays and therefore hold less negative illness perceptions.

Longitudinal analyses were performed in a small sample of *n* = 27 children. Results show that children in acute treatment reported more symptoms over the course of one year, while all other illness perceptions remained stable. As this is the first study exploring longitudinal trajectories of illness perceptions in paediatric oncology, we need to fall back on results from adult oncology for comparison. Most studies found that illness perceptions remained relatively stable over six to twelve months [34–36]. It may be that cancer patients develop their individual illness perceptions at the beginning of treatment (prior to T1) and maintain them over time. Longitudinal studies that cover longer periods of time would be interesting to investigate trajectories across childhood, adolescence, and adulthood.

Many previous studies with child and adult cancer patients and survivors have shown that illness perceptions are associated with HRQoL [33, 37–40]. Similarly in our study, self-rated HRQoL correlated with the perception of symptoms associated with the illness. Also, children who perceived more symptoms, a cyclical trajectory, and more negative consequences, experienced lower illness-related HRQoL (subscale of the KINDL-R questionnaire [23]) as proxy-rated by their parents. Questions on this subscale include items such as “During the past week, my child was afraid that the illness might get worse.”, and “[…] my child was sad because of the illness.” Concerning the other constructs used in this study, no significant correlations were found even though we expected them based on theoretical assumptions of the CSM and previous findings in adult and adolescent samples [16, 30]. Concerning our assessment of emotional symptoms, other questionnaires may be more useful than the SDQ [19] in this context.

All interscale correlations that we found were in line with our hypotheses [16, 28, 30], i.e. the more symptoms the children perceived, the more negative was their perception of consequences and associated emotions; the more chronic the children expected their illness to be, the more cyclical they perceived it and the more negative consequences they expected; and the more negative consequences they expected, the more negative emotions they experienced in association with their illness.

Findings on internal consistency in our study were mixed: While the dimensions “timeline-acute/chronic” and “personal control” showed acceptable internal consistency, Cronbach’s alpha values for the other dimensions were below an acceptable threshold. In regard to the dimension “timeline-cyclical”, this is not surprising, as other studies investigating psychometric properties of the IPQ-R found the same [6, 16, 28]. The low internal consistency of the other scales may be due to different factors: Firstly, the size of alpha depends on the number of items, with fewer items leading to a lower alpha value [41]. Also, some items were answered in the affirmative or negative by almost all children (e.g. item 23), which may lead to lower consistency in this dimension. Furthermore, our sample size was smaller than in the other studies mentioned above, which may also have an impact on internal consistency. While the partly poor internal consistency of the dimensions may be considered a drawback of the puppet interview, one of its benefits is the close relation to the established IPQ-R that allows for the direct comparison of items and dimensions between children and their parents. This comparison then may be used for the adaptation of tailored family-centred psychosocial interventions.

### Strengths and limitations

The puppet interview is an innovative instrument for interviewing very young patients in particular, whose self-report would otherwise not be ascertainable. Due to its playful character the access to the patients is easy. The positive feedback from the children and the clinical experience of the interviewer make it clear that the puppet interview as a diagnostic tool is very well applicable, comprehensible, economical, and practicable, especially in younger children. Furthermore, the puppet interview offers the possibility to derive a play-therapeutic intervention from the diagnostic method using the same methodology. Further research should address the development and evaluation of such an intervention. A limitation of the puppet interview, however, is that a prior familiarization with the methodology is indispensable, which surpasses the mere reading of the manual.

The sample size for psychometric analyses was relatively small (*N* = 75). It allowed for a first exploration of known-groups validity, construct validity, longitudinal trajectories, interscale correlations, and internal consistency. As illness perceptions may differ between age groups, gender, or different diagnoses, subsample analyses of these psychometric properties should be performed in future studies with larger samples and cell count in contingency tables.

Construct validity analyses were performed using proxy-report measures of child’s control beliefs, self-efficacy beliefs, optimism, emotional problems, and HRQoL. Whenever possible, child’s self-report should be used as it may be more accurate. For young children under the age of six years, however, self-report measures on the above-mentioned constructs are rare. In order to obtain one consistent measure, we therefore opted for the parent’s proxy-report. Future studies may also include valid proxy-report measures for specific child’s emotional problems instead of the generic score from the SDQ [19], such as the Depression Inventory for Children and Adolescents (DIKJ [42]).

Finally, as the dimension “treatment control” has been added later to the IPQ-R-Puppet Interview, its psychometric properties could not have been investigated so far. This should be done in a future study.

### Clinical implications

Overall, this study demonstrates the clinical importance of illness perceptions in the psychosocial care of children with cancer, both in acute care and (long-term) follow-up. With the puppet interview, a self-report on illness perceptions in children from the age of four years is possible. This is especially useful as previous research has shown that parents may perceive their child’s well-being and cognitive appraisal differently from the child themselves, e.g. parents often overestimate symptom prevalence in their child [12, 43]. This dissimilarity may be due to the “lens” through which parents view their children, as parent’s own well-being may influence their appraisal of their child. Asking the child themselves about their views and symptoms, on the other hand, may increase their sense of control as well have implication for the administration of medication.

Due to their importance for individual coping with the illness, illness perceptions should also be assessed in other chronic and severe paediatric illnesses. This is now possible with the newly developed puppet interview. We provide this innovative instrument to the international community in order to stimulate research on illness perceptions in young children with different illnesses. Also, the puppet interview may be further adapted as an intervention tool itself: For example, the puppet interview can also help to discover the reasons for lack of cooperation in therapy (e.g. refusal to take medication) and increase compliance. Future studies are intended to develop interventions for deficits found in the individual dimensions of the puppet interview.

### Supplementary Information

**Additional file1:** **Appendix 1.** Interview Guide for the IPQ-R-Puppet Interview.

## Data Availability

The data and materials that support the findings of this study are available from the corresponding author (KH) upon reasonable request.

## Abbreviations

ASKU: Short scale to measure general self-efficacy beliefs
BPI: Berkeley Puppet Interview
CIPQ: Children’s Illness Perception Questionnaire
CSM: Common Sense Model of Illness Representation
DIKJ: Depressions-Inventar für Kinder und Jugendliche (depression inventory for children and adolescents)
DRKS: Deutsches Register Klinischer Studien (German Clinical Trials Register)
IE-4: Short scale to measure internal and external control beliefs
IPQ-R: Illness Perception Questionnaire Revised
HRQoL: Health-related quality of life
PRO: Patient-reported outcome
SDQ: Strengths and Difficulties Questionnaire
SOP-2: Short scale to measure optimism and pessimism
YOU-IPQ-R: Youth Illness Perception Questionnaire Revised
