# Supplementary material for: The puppet interview to measure illness perceptions in paediatric oncology: development and psychometric properties in acute treatment and follow-up care
Source: BMC Pediatr. 2024 Feb 13;24:112. doi: 10.1186/s12887-024-04586-5 (PMC10863186; doi:10.1186/s12887-024-04586-5)
Supplement: Supplementary file 1 — Additional file1: Appendix 1. Interview Guide for the IPQ-R-Puppet Interview. [file 12887_2024_4586_MOESM1_ESM.docx]

# Appendix 1: Interview Guide for the IPQ-R-Puppet Interview

| Study code | _______________ | Gender | male  female  diverse | |  |
| --- | --- | --- | --- | --- | --- |
| Date of interview | _______________­­­­­­­ | Age | | _________ years | |

### Preparation & information about the puppet interview

The puppet interview can be used by researchers as part of a research project or by a case manager (psychosocial staff) in clinical practice as an assessment method.

In case of a research project, the interviewer should make an appointment with the family before meeting with them as the time at the hospital may already be quite stressful. Therefore, the family already knows that they meet with a researcher to receive information about potential participation in a research project. At the information appointment, the child and at least one caregiver/parent should be attending. In addition to verbally informing the family about the research project, the interviewer should present written information about the research project at hand that remain with the family and should contain information about content of the project, duration, potential side effects (e.g., tiredness), contact information, and data privacy rights. The specific content of the information may vary by location. It is useful to also have child-specific written information at hand.

Example for verbal information for the child: “Hello [name]. My name is […]. I work here at the hospital as a researcher and I want to find out how children deal with an illness such as yours. For this, I would like to ask you if you were willing to participate in an interview with two colleagues of mine. Their names are Beppi and Seppi, and they also spent some time in hospital, just like you. Beppi and Seppi have been very curious about you and would like to meet you. They would like to talk to you about your experiences here in the hospital and with your illness and share their own experiences. Would you agree to participate in the interview? The interview would be approximately 15-20 minutes in length and you can take breaks at any point.”

Afterwards, the family should be given time to consider and ask any questions.

### Conducting the interview

Parents should ideally not be present for the interview as this might distract the child. Some children, however, may feel more comfortable with the parents being present. In this case, it is recommended that the parents stay outside of their child’s field of vision and occupy themselves. Parents should furthermore be instructed not to answer on behalf of their child or intervene in the interview in any other way.

The introduction of the puppets may of course be modified. It is important to awaken the child’s interest, e.g., by telling a silly story.

Example for introduction of the hand puppets: “Hi [name]! I would like to introduce you to two colleagues of mine. They currently sleep in this bag over here (point out fabric bag) because they have already played hide-and-seek the whole morning. So, we need to wake them up first. How would you suggest we wake them up? (if necessary, make suggestions, e.g., tapping the bag, singing, shouting) Wake up, Beppi and Seppi, wake up! (puppets “wake up” yawning and come out of the bag).”

| Left hand: Beppi | Right hand: Seppi |
| --- | --- |
| Hi [name]! My name is Beppi. | And I am Seppi. Nice to meet you! |
| Blimey, I was sooooo tired just now because Seppi and I played hide-and-seek together. | I am really bad at hide-and-seek, Beppi always finds me! Do you like hide-and-seek, [name]? What other games do you like? |
| … |  |

As soon as a rapport between the puppets and the interviewer is established, the IPQ-R-Puppet Interview can start:

| You know what, [name]? Last year, I was ill for quite a while and was in hospital. | Me too. |
| --- | --- |
| The doctor said that I had cancer and this way why I had to spend a couple of months at the hospital. | Hm, might be. I also had an illness, but I don’t quite know the name of it. |
| We would like to tell you how we experienced our illness and the time at the hospital. | And we would also like to know about your own experiences and feelings with your illness. Alright? |
| [wait for reaction] |  |
| If you feel the same as I do, then you can tap me or say my name – Beppi. | And if you feel the same as I do, then you can tap me or say my name – Seppi. |
| Shall we give this a try?  [wait for reaction] |  |
| I like vanilla ice cream more than chocolate ice cream. | And I, however, like chocolate ice cream more than vanilla ice cream. What about you, [name]? |
| [check if child understood the instruction] |  |
|  | Alright, let’s start. |

### Symptoms

If the child answers “yes” (1) on a symptom, a follow-up question is asked: “And do you think [symptom] was due to your illness or treatment (if necessary, ask in more detail, e.g., due to your chemotherapy)?”

| No. | Left hand: Beppi | Right hand: Seppi | If yes…. |
| --- | --- | --- | --- |
|  |  | Let’s talk first about the time we were at the hospital because of our illness. | (1) Symptom due to illness/treatment  (0) Symptom **not** due to illness/treatment |
| 1 | (1) Something hurt me. | (0) Nothing hurt me. | (1)  (0) |
| 2 | (1) I had a sore throat. | (0) I didn't have a sore throat. | (1)  (0) |
| 3 | (0) I didn't feel sick. | (1) I felt sick sometimes. | (1)  (0) |
| 4 | (1) I had a hard time breathing. | (0) I was able to breathe quite well. | (1)  (0) |
| 5 | (0) I weighed just as much as ever. | (1) I lost some weight. | (1)  (0) |
| 6 | (0) I didn’t feel tired and exhausted. | (1) I always felt so tired and exhausted. | (1)  (0) |
| 7 | (1) My arms and legs were hurting. | (0) My arms and legs didn't hurt. | (1)  (0) |
| 8 | (0) My eyes didn't sting. | (1) My eyes stung (they were very red). | (1)  (0) |
| 9 | (1) I couldn't breathe in deeply. | (0) I could breathe deeply. | (1)  (0) |
| 10 | (1) I had a headache. | (0) I did not have a headache. | (1)  (0) |
| 11 | (1) My belly hurt. | (0) My belly did not hurt. | (1)  (0) |
| 12 | (1) I could not sleep well. | (0) I could not sleep badly. | (1)  (0) |
| 13 | (0) I wasn’t dizzy. | (1) I was dizzy. | (1)  (0) |
| 14 | (0) I didn’t feel weak. | (1) I felt weak. | (1)  (0) |

### Break

Especially for younger children it may be appropriate to take a short break at this point. The interviewer could use this break to tell a funny story (via the puppets), play a game (e.g., Dobble), or draw with the child.

| Left hand: Beppi | Right hand: Seppi |
| --- | --- |
| Well done, [name]! | Would you like to take a short break and play [game] with us? |
| … |  |
| You know, [name], yesterday Seppi and I talked a lot about what we think about our illnesses. We are curious what you think about your illness. Can we talk a little bit more about this with you?  [wait for reaction] |  |

### Other IPQ-R dimensions

After each thesis and antithesis, one of the puppets asks the child “How about you, [name]?”

| No. | Left hand: Beppi | Right hand: Seppi |
| --- | --- | --- |
| 15 | (1) My cancer illness lasts a long time. | (0) My cancer illness does not last long. |
| 16 | (0) There were little changes because of my cancer illness - I still get to do everything I enjoy. | (1) My cancer illness caused many changes - I'm no longer allowed to do everything I enjoy. |
| 17 | (0) I can't do anything to feel better. | (1) I can do a lot to feel better. |
| 18 | (0) My cancer illness is a mystery to me. | (1) My cancer illness isn't a mystery to me. |
| 19 | (1) When something hurts me, it’s always the same thing. | (0) When something hurts me, they are different things. |
| 20 | (0) I am not sad because of my cancer illness. | (1) I am sad because of my cancer illness. |
| 21 | (1) My treatment (e.g., chemotherapy, radiotherapy, surgery) will make my cancer illness go away. | (0) My treatment (e.g., chemotherapy, radiotherapy, surgery) will not make my cancer illness go away. |
| 22 | (1) I'm sure I'll be ill for a long time. | (0) I'm sure I'll only be ill for a short time. |
| No. | Left hand: Beppi | Right hand: Seppi |
| 23 | (0) Some children don't mind my cancer illness - they still play with me. | (1) Other children are bothered by my cancer illness - they no longer play with me. |
| 24 | (0) I can't do anything about my cancer illness. | (1) I can do something about my cancer illness. |
| 25 | (1) I understand my cancer illness. | (0) I don't understand my cancer illness. |
| 26 | (1) I don't know how I will feel tomorrow. | (0) I know how I will feel tomorrow. |
| 27 | (0) When I think about my cancer illness, I stay quite calm. | (1) When I think about my cancer illness, it makes me upset. |
| 28 | (1) My treatment helps me feel less sick. | (0) My treatment does not help me feel less sick. |
| 29 | (1) I will have my cancer illness for a very long time. | (0) I won't have my cancer illness for long, it will be gone soon. |
| 30 | (0) My cancer illness doesn't cause any problems and worries to my parents. | (1) My cancer illness causes problems and worries for my parents. |
| 31 | (0) I can't do anything myself to make myself feel better. | (1) I can do something myself to feel better. |
| 32 | (1) I understand why my cancer illness is there. | (0) I don’t understand why my cancer illness is there. |
| 33 | (1) Due to my cancer illness, I sometimes feel better and sometimes worse. | (0) I always feel the same due to my cancer illness. |
| 34 | (1) My cancer illness makes my anxious. | (0) My cancer illness doesn’t make me anxious. |
| 35 | (1) My treatment can control my cancer illness | (0) My treatment cannot control my cancer illness. |

### Evaluation and wrap-up

| Left hand: Beppi | Right hand: Seppi |
| --- | --- |
| Now we have almost reached the end of our interview.  We are curious how you liked talking to us: |  |
| (1) I understood all the questions well. | (1) I, however, had trouble understanding some of the questions. What about you, [child]?  (If the child also had trouble understanding some of the questions, it is recommended to ask them which questions they did not understand. Oftentimes, they meant the questions that they had already inquired about during the interview and that could be clarified in this way.) |
| (1) And I enjoyed our interview. | (0) I didn't enjoy our conversation that much.  (If the child inquires after reasons for this, Seppi could, for example, answer that some of the questions reminded him about his own illness which made him sad.) |
| All right, we've now asked you all the questions we wanted to know. | Well done!  Now that we've peppered you with so many questions, do you want to ask us anything else? |
| … |  |

To wrap up, either the puppets tell the child that they need to leave due to another appointment, or that they are tired again and want to return to sleep. Some children may want to continue playing or drawing with the interviewer or the puppets.

### Behaviour observation (e.g., timid) and interview setting (e.g., hospital room, interruptions)

|  |
| --- |

### Analysis (with SPSS)

##### Symptoms dimension (“identity”)

This dimension is differentiated in “symptoms experienced during the illness/treatment” and “symptoms experienced and caused by the illness/treatment”. The latter is known as the symptoms/identity dimension.

The variable “ipq_e_symptom” for each of the 14 symptoms has the expression 0 (if the symptom has not been experienced) or 1 (if the symptom has been experienced).

The variable “ipq_c_symptom” for each of the 14 symptoms has the expression 0 (either if the symptom has not been experienced, or if the symptom has been experienced but not caused by the illness/treatment) or 1 (if the symptom has been experienced and caused by the illness/treatment).

The sum score “identity_experienced” and “identity_caused” can take on values between [0, 14].

COMPUTE identity_experienced=SUM(ipq_e_pain, ipq_e_throat, ipq_e_nausea, ipq_e_breathless, ipq_e_weight, ipq_e_exhaustion, ipq_e_joints, ipq_e_eyes, ipq_e_breathshort, ipq_e_headache, ipq_e_digeston, ipq_e_sleep, ipq_e_vertigo, ipq_e_asthenia)

EXECUTE.

COMPUTE identity_caused=SUM((ipq_c_pain, ipq_c_throat, ipq_c_nausea, ipq_c_breathless, ipq_c_weight, ipq_c_exhaustion, ipq_c_joints, ipq_c_eyes, ipq_c_breathshort, ipq_c_headache, ipq_c_digeston, ipq_c_sleep, ipq_c_vertigo, ipq_c_asthenia)

EXEXUTE.

##### Other dimensions (timeline-acute/chronic, timeline-cyclical, consequences, personal control, treatment control, coherence, emotional representations)

Each of the variables “ipq_15” to “ipq_35” has the expression 0 or 1. The sum score for each dimension therefore ranges from [0, 3]. Higher scores indicate a more negative perception of chronicity, cyclicity, and emotional representations, and more positive perception of personal control, treatment control, and illness coherence.

COMPUTE timeline=SUM(ipq_15, ipq_22, ipq_29)

COMPUTE timecycle=SUM(ipq_19, ipq_26, ipq_33)

COMPUTE consequences=SUM(ipq_16, ipq_23, ipq_30)

COMPUTE personalcontrol=SUM(ipq_17, ipq_24, ipq_31)

COMPUTE treatmentcontrol=SUM(ipq_21, ipq_28, ipq_35)

COMPUTE coherence=SUM(ipq_18, ipq_25, ipq_32)

COMPUTE emotion=SUM(ipq_20, ipq_27, ipq_34)

EXECUTE.
